# Co-administration of Favipiravir and the Remdesivir Metabolite GS-441524 Effectively Reduces SARS-CoV-2 Replication in the Lungs of the Syrian Hamster Model

**DOI:** 10.1128/mbio.03044-21

**Published:** 2022-02-01

**Authors:** Shiho Chiba, Maki Kiso, Noriko Nakajima, Shun Iida, Tadashi Maemura, Makoto Kuroda, Yuko Sato, Mutsumi Ito, Moe Okuda, Shinya Yamada, Kiyoko Iwatsuki-Horimoto, Tokiko Watanabe, Masaki Imai, Tammy Armbrust, Ralph S. Baric, Peter J. Halfmann, Tadaki Suzuki, Yoshihiro Kawaoka

**Affiliations:** a Influenza Research Institute, Department of Pathobiological Sciences, School of Veterinary Medicine, University of Wisconsin-Madison, Wisconsin, USA; b Division of Virology, Department of Microbiology and Immunology, Institute of Medical Science, University of Tokyo, Tokyo, Japan; c Department of Pathology, National Institute of Infectious Diseases, Tokyo, Japan; d Department of Molecular Virology, Research Institute for Microbial Diseases, Osaka University, Osaka, Japan; e Department of Epidemiology, Gillings School of Global Public Health, University of North Carolina at Chapel Hill, Chapel Hill, North Carolina, USA; University of Maryland School of Medicine; Johns Hopkins Bloomberg School of Public Health

**Keywords:** SARS-CoV-2, favipiravir, remdesivir, GS-441524, Syrian hamster

## Abstract

Severe acute respiratory syndrome coronavirus 2 (SARS-CoV-2) has spread worldwide since December 2019, causing coronavirus disease 2019 (COVID-19). Although vaccines for this virus have been developed rapidly, repurposing drugs approved to treat other diseases remains an invaluable treatment strategy. Here, we evaluated the inhibitory effects of drugs on SARS-CoV-2 replication in a hamster infection model and in *in vitro* assays. Favipiravir significantly suppressed virus replication in hamster lungs. Remdesivir inhibited virus replication *in vitro*, but was not effective in the hamster model. However, GS-441524, a metabolite of remdesivir, effectively suppressed virus replication in hamsters. Co-administration of favipiravir and GS-441524 more efficiently reduced virus load in hamster lungs than did single administration of either drug for both the prophylactic and therapeutic regimens; prophylactic co-administration also efficiently inhibited lung inflammation in the infected animals. Furthermore, pretreatment of hamsters with favipiravir and GS-441524 effectively protected them from virus transmission via respiratory droplets upon exposure to infected hamsters. Repurposing and co-administration of antiviral drugs may help combat COVID-19.

## INTRODUCTION

The novel coronavirus severe acute respiratory syndrome coronavirus-2 (SARS-CoV-2) was first reported in humans in China in December 2019, and it spread across the world within several months, causing coronavirus disease 2019 (COVID-19). As of October 2021, the virus has continued to spread worldwide, and several different variants have emerged which are antigenically advanced and/or have greater replication fitness than the original virus. More than 4.5 million global deaths have been reported to date. Several vaccines with different modalities have been approved and rapidly distributed across numerous countries; however, given that escape variants continue to appear, there remains an urgent need to establish COVID-19 treatment regimens by repurposing drugs currently approved to treat other infections or diseases. Several drugs, including favipiravir and remdesivir, which are approved for the treatment of other diseases have been tested for efficacy against SARS-CoV-2 in clinical trials (1–7). For remdesivir, some reports, including meta-analyses, have shown marked clinical improvements, such as shorter recovery times, with remdesivir treatment compared to placebo (4, 6, 8, 9), although statistical significance was not always achieved (4, 5, 7). It is important to note that clinical trials such as these are limited in that they are generally not designed to evaluate the effects of drugs on virus load in patients under different conditions. In contrast, animal models, in which various experimental conditions can be tested, can advance our understanding of the effectiveness of potential drug therapies. Syrian hamsters are highly susceptible to the virus and an excellent model of SARS-CoV-2 infection (10–15). Utilizing this model, investigators have shown that favipiravir administration inhibits virus replication and inflammation in the lungs and have examined the pharmacokinetics of the drug *in vivo* (13, 14). In the direct-contact transmission model, high-dose favipiravir pretreatment of a naive animal that was then co-housed with an infected animal significantly reduced the virus titers in the lungs (14). Here, we found that co-administration of favipiravir and GS-441524, a metabolite of remdesivir, more potently reduced virus load and inflammation in the lungs of infected hamsters than either favipiravir or GS-441524 alone. Furthermore, by using a setup that allows only airborne transmission without direct contact between animals, we demonstrated that co-administration of these drugs to naive animals protected them, albeit not completely, from airborne virus transmission. These results suggest the feasibility of co-administering these repurposed drugs as a treatment option for COVID-19.

## RESULTS

### Effects of approved drugs on SARS-CoV-2 replication in the hamster model and *in vitro*.

We first examined whether drugs that are currently approved for COVID-19 or other diseases have inhibitory effects on SARS-CoV-2 replication in Syrian hamsters. One day prior to SARS-CoV-2 challenge (day –1), hamsters were prophylactically administered favipiravir, lopinavir/ritonavir, nelfinavir, hydroxychloroquine sulfate, remdesivir (GS-5734), ciclesonide, nafamostat mesylate, ivermectin, mefloquine, umifenovir, or cepharanthine (see regimens in Table S1 in the supplemental material), and were then intranasally infected with 10 PFU/animal of SARS-CoV-2 (day 0). Body weight was monitored daily as a clinical symptom marker, and on day 4 postinfection, lungs and nasal turbinates were sampled to examine infectious virus titers in plaque assays. Administration of lopinavir/ritonavir, nelfinavir, hydroxychloroquine sulfate, remdesivir (GS-5734), ciclesonide, nafamostat mesylate, ivermectin, mefloquine, umifenovir, or cepharanthine did not significantly affect virus replication in these organs, or body weights, under these experimental conditions (Fig. S1A–X); the findings for hydroxychloroquine sulfate and lopinavir/ritonavir are consistent with previous studies in hamster and mouse models, respectively (14, 16, 17).

10.1128/mBio.03044-21.1 TABLE S1 Dosage regimens in Syrian hamster models. *, p.o., oral gavage; s.c., subcutaneous inoculation; i.n., intranasal inoculation; i.p., intraperitoneal inoculation. **, Animals were infected with virus on day 0. Download Table S1, DOCX file, 0.01 MB. Copyright © 2022 Chiba et al. 2022 Chiba et al. https://creativecommons.org/licenses/by/4.0/ This content is distributed under the terms of the Creative Commons Attribution 4.0 International license.

10.1128/mBio.03044-21.4 FIG S1 Inhibitory effect of drugs on SARS-CoV-2 replication in the lungs and nasal turbinate of hamsters. Each group of hamsters (*n* = 4) was administered drug or vehicle control as detailed in Table S1, beginning on the day before virus challenge (day –1). The hamsters were then intranasally inoculated with 10 PFU of SARS-CoV-2 (day 0). Infectious virus titers in the lungs and nasal turbinate (PFU/g) were examined by performing plaque assays (panels A, B, D, E, G, H, J, K, M, N, P, Q, S, T, V, and W; dots and bars show the value for each animal and the average in each group, respectively [*n *= 4, mean ± SD]). Body weights (compared to those on day 0) of hamsters inoculated with SARS-CoV-2 (*n* = 4, mean ± SD) were monitored from day 0 to day 4 (panels C, F, I, L, O, R, U, X). Download FIG S1, TIF file, 1.4 MB. Copyright © 2022 Chiba et al. 2022 Chiba et al. https://creativecommons.org/licenses/by/4.0/ This content is distributed under the terms of the Creative Commons Attribution 4.0 International license.

**FIG 1:**
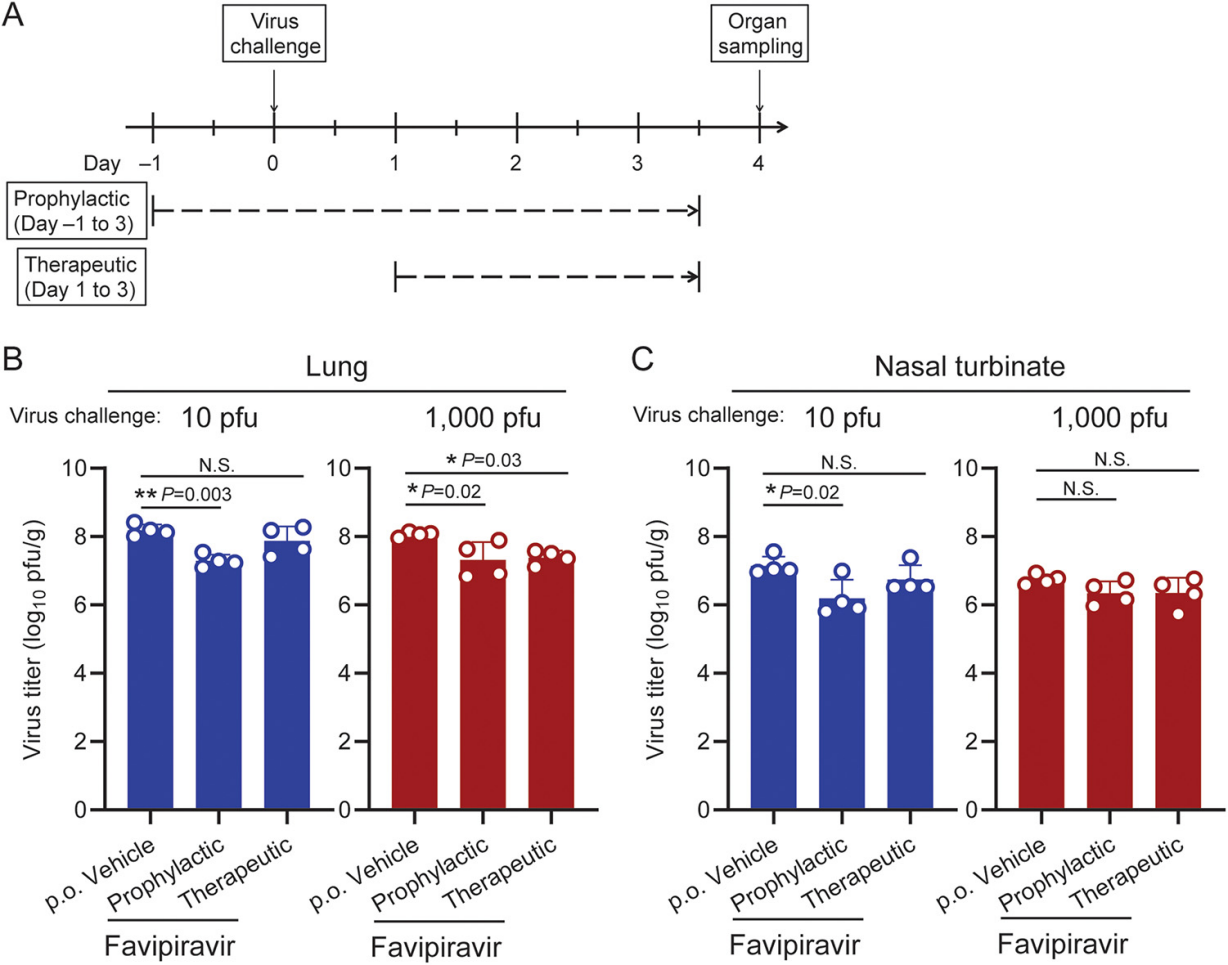
Inhibitory effects of favipiravir on SARS-CoV-2 replication in the lungs and nasal turbinate of hamsters. (A) Timeline of favipiravir administration and virus challenge schedule in the hamster infection model. (B, C) Each group of hamsters (*n* = 4) was administered vehicle control or favipiravir (300 mg/kg) as a prophylactic (days –1 to 3; twice/day) or therapeutic regimen (days 1 to 3; twice/day). The hamsters were intranasally inoculated with 10 or 1,000 PFU of SARS-CoV-2 (day 0). Infectious virus titers (PFU/g) in the lungs (panel B) and nasal turbinate (panel C) were examined by performing plaque assays. Dots and bars show the value for each animal and the average in each group, respectively (*n* = 4, mean ± SD [standard deviation of the mean]). *, *P* < 0.05; NS, not significant; determined by a one-way analysis of variance (ANOVA) and corrected for multi-group comparison using Dunnett’s test.

In contrast, favipiravir, which was first approved as an antiviral for influenza virus treatment in Japan, significantly reduced virus replication in the lungs of hamsters challenged with a low (10 PFU) or high dose (1,000 PFU) of SARS-CoV-2 (Fig. 1A and B; the mean virus load was reduced to 12.8% or 29.2% of the vehicle control group, with 10 or 1,000 PFU of challenge virus, respectively) when given as a prophylactic regimen (dosages on days –1 to 3: 300 mg/kg, twice/day). This is consistent with the findings of a previous study in hamsters (13, 14) and with findings in human clinical trials, which indicated that favipiravir can reduce recovery times for mild-to-moderate patients (3). Therapeutic administration of favipiravir (dosages on days 1 to 3: 300 mg/kg, twice/day) also reduced virus titers in the lungs after a high-dose challenge (mean titer reduced to 22.4% of the control group), although not significantly after the lower dose (mean titer reduced to 62.9% of the control group; Fig. 1B). Virus titers in the nasal turbinate were also slightly reduced, although a statistically significant difference was seen only with the therapeutic regimen and low-dose virus challenge (mean titer reduced to 18.9% of the control group; Fig. 1C). Body weight changes were not improved by favipiravir treatment (Fig. S2) under these conditions.

10.1128/mBio.03044-21.5 FIG S2 Body weight change after SARS-CoV-2 with favipiravir administration. Each group of hamsters (*n* = 4) was administered favipiravir (300 mg/kg; twice/day), in either a prophylactic or a therapeutic regimen, or vehicle control. The hamsters were then intranasally inoculated with 10 PFU (panel A) or 1,000 PFU (panel B) of SARS-CoV-2 (day 0). Body weights (compared to those on day 0; *n* = 4, mean ± SD) were monitored from days 0 to 4. Download FIG S2, TIF file, 0.7 MB. Copyright © 2022 Chiba et al. 2022 Chiba et al. https://creativecommons.org/licenses/by/4.0/ This content is distributed under the terms of the Creative Commons Attribution 4.0 International license.

To examine the inhibitory effects of these drugs on SARS-CoV-2 replication *in vitro*, we determined half-maximal effective concentrations (EC_50_) and half-maximal inhibitory concentrations (IC_50_) (Table S2). Remdesivir effectively inhibited cytopathic effects (CPE) in VeroE6/TMPRSS2 and Calu-3 cells (EC_50_ = 1.7 μM and 0.3 μM, respectively), with 2- to 3-log lower EC_50_ values than those of favipiravir (EC_50_ = 130 μM and 320 μM, respectively), although it did not significantly inhibit virus replication in hamsters (Fig. S1D and E). Nelfinavir, a protease inhibitor approved for the treatment of HIV infection, showed comparable suppressive efficacy to that of remdesivir, with effectiveness at <10 μM under these *in vitro* conditions, despite its lack of efficacy in the hamster model (Fig. S1A and B). These results are consistent with those of some previous studies which have demonstrated the *in vitro* inhibitory efficacy of remdesivir (18–24) or nelfinavir (18, 20, 25–27) on SARS-CoV-2 replication.

10.1128/mBio.03044-21.2 TABLE S2 Inhibitory effects of drugs on SARS-CoV-2 replication *in vitro*. *, Concentration could not be determined at higher concentrations because of cytotoxicity. **, EC_50_, cytopathic effect (CPE) inhibition assay; IC_50_, plaque reduction assay. Download Table S2, DOCX file, 0.01 MB. Copyright © 2022 Chiba et al. 2022 Chiba et al. https://creativecommons.org/licenses/by/4.0/ This content is distributed under the terms of the Creative Commons Attribution 4.0 International license.

### Inhibitory effects of GS-441524 on SARS-CoV-2 replication in the hamster model.

**FIG 2:**
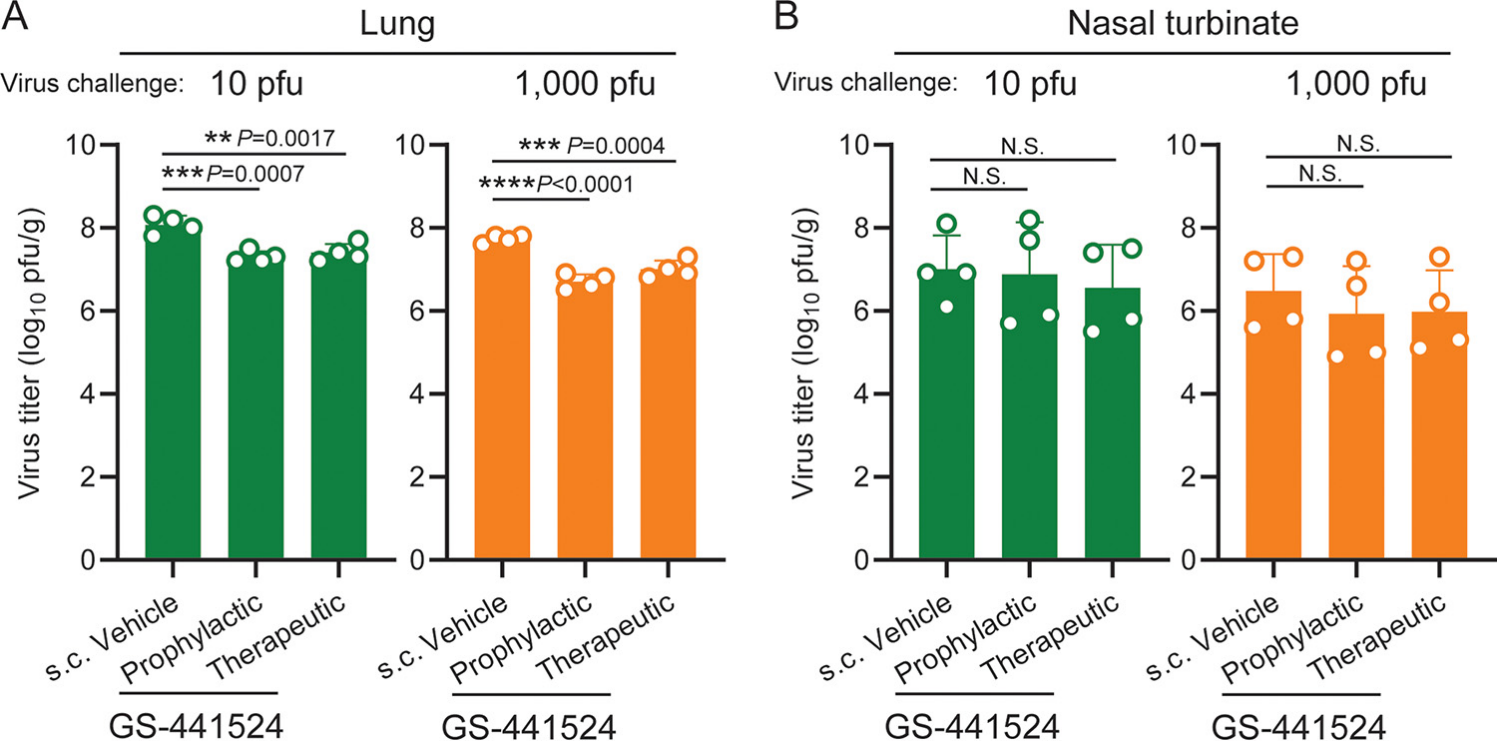
Inhibitory effects of GS-441524 on SARS-CoV-2 replication in the lungs and nasal turbinate of hamsters. (A, B) Each group of hamsters (*n* = 4) was administered vehicle control or GS-441524 (25 mg/kg) as a prophylactic (days –1 to 3; twice/day) or therapeutic regimen (days 1 to 3; twice/day). The hamsters were intranasally inoculated with 10 or 1,000 PFU of SARS-CoV-2 (day 0). Infectious virus titers (PFU/g) in the lungs (panel A) and nasal turbinate (panel B) were examined by performing plaque assays. Dots and bars show the value for each animal and the average in each group, respectively (*n* = 4, mean ± SD). *, *P* < 0.05; NS, not significant; determined by a one-way analysis of variance (ANOVA) and corrected for multi-group comparison using Dunnett’s test.

The inhibitory effects of remdesivir on SARS-CoV-2 replication observed *in vitro* prompted us to evaluate the *in vivo* effects of GS-441524, a metabolite of remdesivir. Although GS-441524 is not currently approved for clinical use for humans, its efficacy has been shown for feline coronavirus (28, 29). Hamsters were administered GS-441524 either prophylactically (days –1 to 3: 25 mg/kg, twice/day) or therapeutically (days 1 to 3: 25 mg/kg, twice/day), or were administered vehicle control, and were then infected with SARS-CoV-2 on day 0. Infectious viral load in the lungs on day 4 postinfection was significantly reduced by both prophylactic and therapeutic regimens in both low-dose (10 PFU) and high-dose (1,000 PFU) virus infections (Fig. 2A), With the prophylactic regimen, mean viral loads in the lungs were reduced to 15.9% or 9.9% of that of the vehicle control group with low- or high-dose virus infection, respectively; with the therapeutic regimen, mean viral loads were reduced to 21.3% or 20.4% of that of the vehicle control group with low- or high-dose virus infection, respectively. Virus replication in the nasal turbinate and body weight loss were not significantly affected by the treatment (Fig. 2B, Fig. S3).

10.1128/mBio.03044-21.6 FIG S3 Body weight change after SARS-CoV-2 with GS-441524 administration. Each group of hamsters (*n* = 4) was administered GS-441524 (25 mg/kg; twice/day), either prophylactically or therapeutically, or vehicle control. The hamsters were then intranasally inoculated with 10 PFU (panel A) or 1,000 PFU (panel B) of SARS-CoV-2 (day 0). Body weights (compared to those on day 0; *n* = 4, mean ± SD) were monitored from days 0 to 4. Download FIG S3, TIF file, 0.6 MB. Copyright © 2022 Chiba et al. 2022 Chiba et al. https://creativecommons.org/licenses/by/4.0/ This content is distributed under the terms of the Creative Commons Attribution 4.0 International license.

### Co-administration of GS-441524 and favipiravir suppresses SARS-CoV-2 replication *in vivo*.

**FIG 3:**
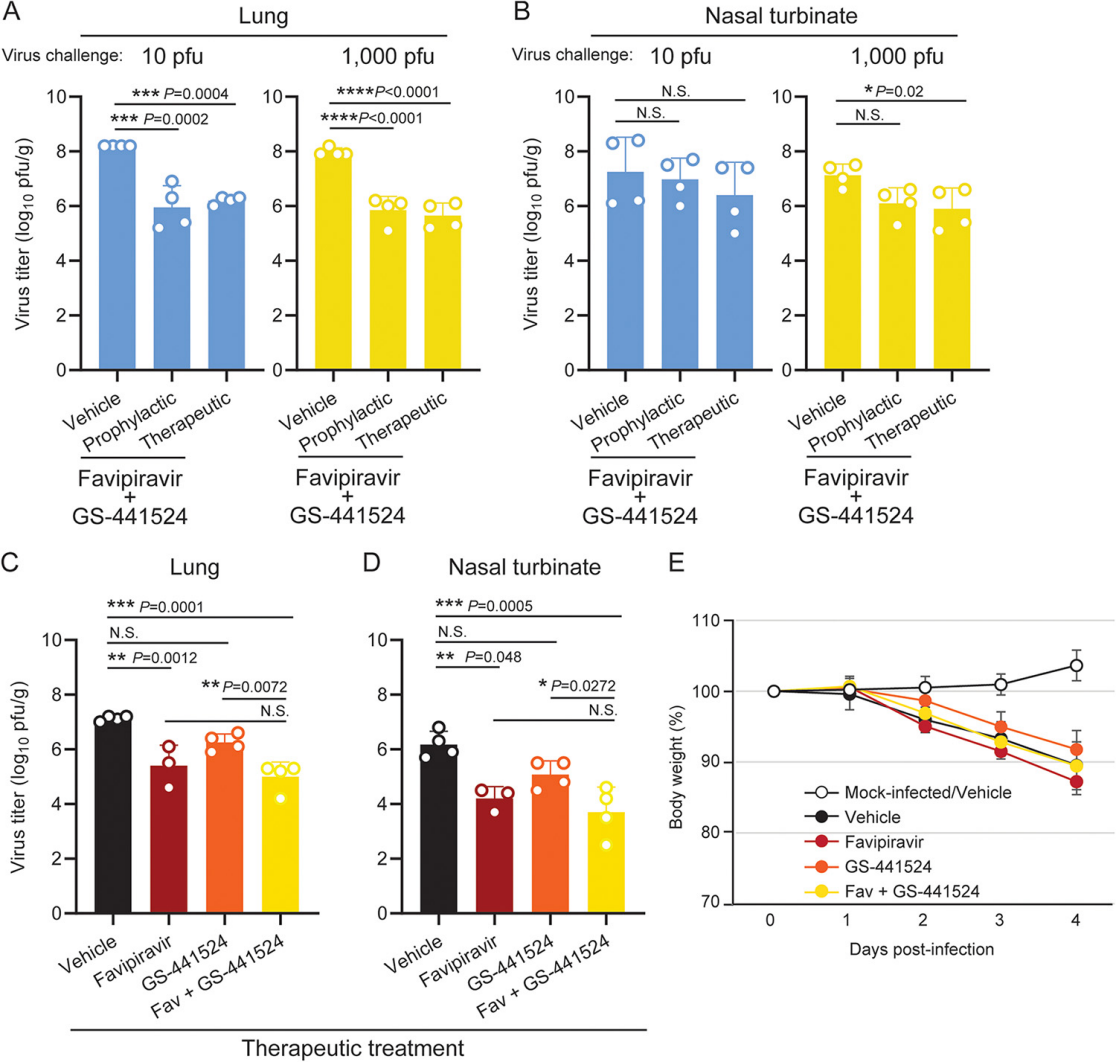
Inhibitory effects of combination of GS-441524 and favipiravir on SARS-CoV-2 replication in the lungs and nasal turbinate of hamsters. (A, B) Each group of hamsters (*n* = 4) was administered vehicle control or the combination of favipiravir (300 mg/kg) and GS-441524 (25 mg/kg) as a prophylactic regimen (days –1 to 3; twice/day) or a therapeutic regimen (days 1 to 3; twice/day). The hamsters were intranasally inoculated with 10 or 1,000 PFU of SARS-CoV-2 (day 0). Infectious virus titers (PFU/g) in the lungs (panel A) and nasal turbinate (panel B) were examined by performing plaque assays. (C to E) Each group of hamsters (*n* = 3 for favipiravir treatment; *n* = 4 for other groups) was mock-infected or infected with 1,000 PFU of SARS-CoV-2 (day 0), and was administered vehicle control, favipiravir (300 mg/kg) GS-441524 (25 mg/kg), or a combination of both drugs as a therapeutic regimen (days 1 to 3; twice/day). Infectious virus titers (PFU/g) in the lungs (panel C) and nasal turbinate (panel D) were examined by performing plaque assays. Body weight change was monitored for 4 days (panel E; mean ± SD). Dots and bars in panels A to D show the value for each animal and the average in each group, respectively (mean ± SD). *, *P* < 0.05; NS, not significant; determined by a one-way analysis of variance (ANOVA) and corrected for multi-group comparison using Dunnett’s test.

We also tested co-administration of favipiravir and GS-441524 in the same hamster model. Both the prophylactic (days –1 to 3; twice/day) and the therapeutic (days 1 to 3; twice/day) co-administration regimens inhibited virus replication in the lungs more drastically than either favipiravir or GS-441524 alone, reducing the viral loads on day 4 by 2 log-fold compared to those in the vehicle control-treated animals (Fig. 3A). Virus replication in the nasal turbinates was also inhibited by prophylactic or therapeutic treatment in the high-dose virus challenge, though there was not always significant difference (Fig. 3B). Body weight was not affected by treatment (Fig. S4). To directly compare the effects of favipiravir or GS-441524 treatment alone or in combination side-by-side, we examined the effectiveness of each therapeutic regimen after a 1,000-PFU virus challenge (Fig. 3C–E). Co-administration of favipiravir and GS-441524 reduced virus titers effectively in both the lungs (Fig. 3C) and the nasal turbinate (Fig. 3D) on day 4, although no statistically significant difference was seen compared to the favipiravir-treated group. Body weight change, which may reflect both disease severity and drug toxicity, was not significantly different between the vehicle-treated and drug-treated groups after virus infection (Fig. 3E). To investigate whether the effects of favipiravir and GS-441524 are synergistic, we also tested the antiviral efficacy of the combination treatment in an *in vitro* plaque reduction assay (Fig. S5). Although GS-441524 alone almost completely inhibited plaque formation at 3 μM, favipiravir alone did not show an antiviral effect within the concentration range that did not cause cytotoxicity (≤300 μM; Fig. S5A), which is consistent with the IC_50_ analysis (Table S2). Synergy scores for the combination treatment were calculated using SynergyFinder (30). The scores within –5 to 5 for all of the drug-dose combinations analyzed, and the summary synergy score of 0.136, suggest that there was no synergy between the two drugs under these *in vitro* conditions (Fig. S5B).

10.1128/mBio.03044-21.7 FIG S4 Body weight change after SARS-CoV-2 with co-administration of favipiravir and GS-441524. Each group of hamsters (*n* = 4) was administered favipiravir (300 mg/kg; twice/day) and GS-441524 (25 mg/kg; twice/day), either prophylactically or therapeutically, or vehicle control. The hamsters were then intranasally inoculated with 10 PFU (panel A) or 1,000 PFU (panel B) of SARS-CoV-2 (day 0). Body weights (compared to those on day 0; *n* = 4, mean ± SD) were monitored from days 0 to 4. Download FIG S4, TIF file, 0.7 MB. Copyright © 2022 Chiba et al. 2022 Chiba et al. https://creativecommons.org/licenses/by/4.0/ This content is distributed under the terms of the Creative Commons Attribution 4.0 International license.

10.1128/mBio.03044-21.8 FIG S5 *In vitro* antiviral activity of the favipiravir and GS-441524 combination against SARS-CoV-2. (A) Dose-response matrix of relative inhibition by the favipiravir and GS-441524 combination on the plaque-forming activity of SARS-CoV-2. Heatmap shows relative viral inhibition scaled to a range of 0% to 100%. Each value is the average of two individual assays. (B) The ZIP synergy score surface plot was calculated and visualized using SynergyFinder v2.0. The plot highlights synergistic and antagonistic dose regions in red and green, respectively. A summary synergy score of around 0 (between –10 and 10) is likely to be additive, whereas a score of >10 is likely to be synergistic, according to SynergyFinder. Download FIG S5, TIF file, 1.0 MB. Copyright © 2022 Chiba et al. 2022 Chiba et al. https://creativecommons.org/licenses/by/4.0/ This content is distributed under the terms of the Creative Commons Attribution 4.0 International license.

### The effect of co-administration of GS-441524 and favipiravir on pathogenicity in lungs.

**FIG 4:**
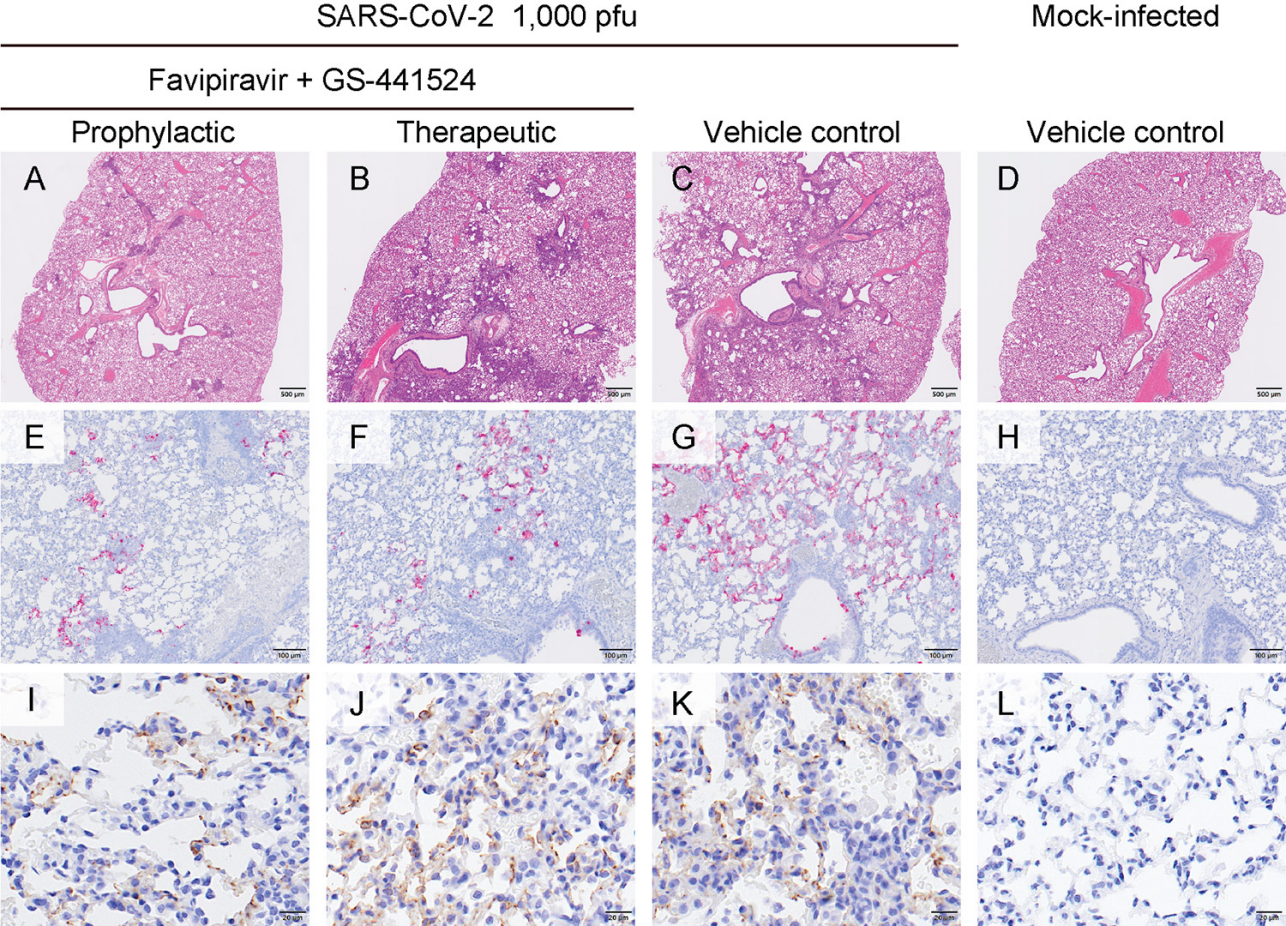
Pathological analysis of hamster lungs. Representative histopathological images of the lungs of hamsters that were mock-infected (panels D, H, L) or intranasally inoculated with 1,000 PFU of SARS-CoV-2 (day 0) and co-administered favipiravir (300 mg/kg) and GS-441524 (25 mg/kg) as a prophylactic (days –1 to 3; twice/day) (panels A, E, I) or therapeutic regimen (days 1 to 3; twice/day) (panels B, F, J), or vehicle control (panels C, G, K). The lungs were analyzed on day 4 postinfection. (E to H) *In situ* hybridization for SARS-CoV-2 viral RNA detection. (I to L) Immunohistochemistry for SARS-CoV-2 viral antigen nucleocapsid protein detection. Panels A to D: scale bar = 500 μm. Panels E to G: scale bar = 100 μm. Panels I to L: scale bar = 20 μm.

To evaluate the effect of co-administration of favipiravir and GS-441524 on lung pathology, lung sections from day 4 were histopathologically analyzed (Fig. 4) and an inflammation score for each pulmonary lobe was determined according to the criteria described in Materials and Methods (Table S3). The lung sections from the therapeutic co-administration group (days 1 to 3: twice daily) and the vehicle control group (Fig. 4B and C, respectively) showed extensive inflammatory cell infiltration, mainly in the peri-bronchial regions; this infiltration was observed in more than 20% of the lung sections, with no obvious difference between the therapeutic co-administration and vehicle control groups. In contrast, the lung sections from the prophylactic co-administration group (days –1 to 3: twice daily) or the mock animals showed only limited numbers of small foci of inflammatory cell infiltration or no inflammation, respectively (Fig. 4A and D, respectively). In the prophylactic and therapeutic co-administration groups, viral RNA signals, which were specifically detected by *in situ* hybridization, were detected only in relatively limited regions of the lung sections, with no obvious difference between the two groups (Fig. 4E and F, respectively). In contrast, in the vehicle control group, viral RNA signals were detected across more extended regions (Fig. 4G) compared to those of the groups treated with either drug regimen (Fig. 4E and F). Notably, immunohistochemistry for SARS-CoV-2 antigen nucleocapsid protein revealed that virus antigens were present in bronchial and alveolar epithelial cells in each group with virus infection, regardless of the presence or absence of drugs or the timing of administration, and there were no clear differences in the number of virus antigen-positive cells between the co-administration groups (prophylactic and therapeutic; Fig. 4I and J, respectively) and the vehicle control groups (Fig. 4K). Taken together, in comparison to the vehicle control group, the prophylactic co-administration group had reduced viral spread and inflammation in the lungs, whereas the therapeutic co-administration group had reduced viral spread in the lungs but no difference in pulmonary inflammation on day 4.

10.1128/mBio.03044-21.3 TABLE S3 Pathological severity scores of the lung tissue sections. Scoring system: 0, no pathological change; 1, affected area (≤10%); 2, affected area (<50%, >10%); 3, affected area (≥50%); an additional point was added when pulmonary edema and/or alveolar hemorrhage was observed. Download Table S3, DOCX file, 0.01 MB. Copyright © 2022 Chiba et al. 2022 Chiba et al. https://creativecommons.org/licenses/by/4.0/ This content is distributed under the terms of the Creative Commons Attribution 4.0 International license.

### Protective efficacy of GS-441524 and favipiravir pretreatment of hamsters in the transmission model.

**FIG 5:**
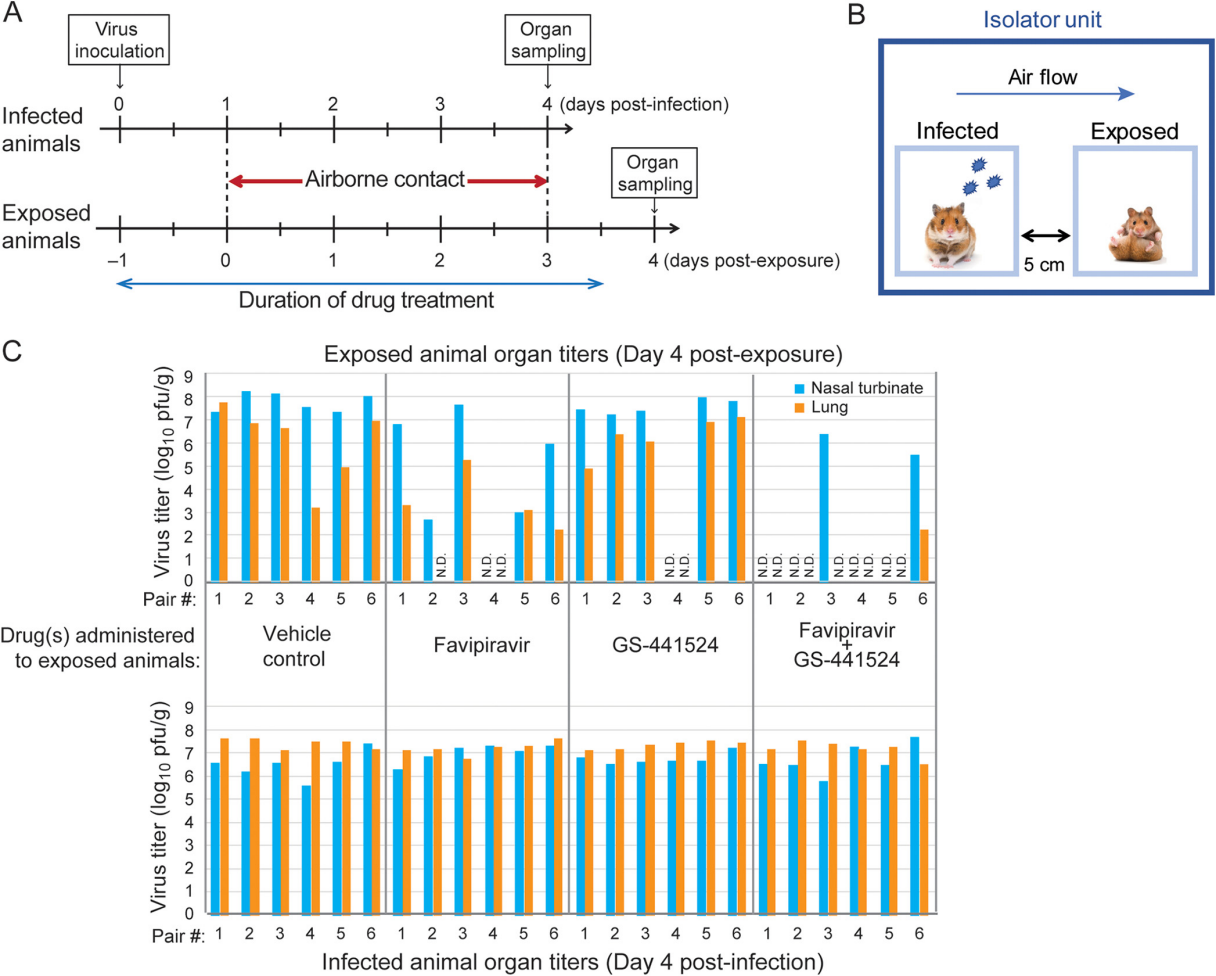
Inhibitory effects of GS-441524 and/or favipiravir on SARS-CoV-2 airborne transmission between hamsters. (A, B) Timeline (panel A) and scheme (panel B) of transmission study. Naive hamsters (exposed animals) were administered favipiravir (300 mg/kg) and/or GS-441524 (25 mg/kg) or vehicle control (days –1 to 3 post-exposure; twice/day; *n* = 6/group). Another group of animals (infected animals) was inoculated with 1,000 PFU of SARS-CoV-2 Spike-D614G variant. One day after the exposed animals began their treatment regimen, each exposed animal was paired with one of the infected animals with a 5-cm gap between their cages. Organs were harvested for virus titration on day 4 postinfection or day 4 post-exposure from infected or exposed animals, respectively. (C) Infectious virus titers (PFU/g) in the nasal turbinate and lungs on day 4 post-exposure (upper panel) or day 4 postinfection (lower panel) were examined by performing plaque assays. ND, not detected (limit of detection: 10 PFU/organ).

Finally, we examined whether pretreatment with GS-441524 and/or favipiravir could protect animals from SARS-CoV-2 infection, using the hamster transmission model (31). Naive hamsters (exposed animals) were administered favipiravir (300 mg/kg) and/or GS-441524 (25 mg/kg), or vehicle control, twice/day (*n* = 6/group); another group of animals (infected animals) was inoculated with 1,000 PFU of SARS-CoV-2 Spike-D614G virus, a variant with the single mutation D614G in the Spike protein, which results in higher transmissibility than that of the original Wuhan virus (31). One day after initiating drug treatment of the exposed animals and virus inoculation of the infected group, each exposed animal was paired with one of the infected animals with a 5-cm gap between their cages, allowing only airborne viral transmission between animals (Fig. 5A and B). On day 4 post-exposure, the exposed animals were euthanized to titrate the virus loads in the nasal turbinate and lungs. The organs of infected animals were also titrated to confirm virus replication on day 4 postinfection (Fig. 5C; lower panel). For the vehicle control group, the nasal turbinate and lungs of all exposed animals showed high virus titers, underscoring the high transmissibility of the virus between the hamsters (Fig. 5C; upper left). With favipiravir administration, the exposed animals showed lower virus loads, especially in the lungs, suggesting that favipiravir contributed to the retardation of transmission and/or inhibition of virus growth (Fig. 5C; upper middle left). With GS-441524 administration, the effect on viral load was more subtle (Fig. 5C; upper middle right). With favipiravir or GS-441524 administration, virus transmission was not detected in 1 pair out of 6. Strikingly, with co-administration of favipiravir and GS-441524, 4 of the 6 exposed animals were protected from virus transmission as of day 4 post-exposure, although this was not statistically significant compared with the vehicle control group (Fig. 5C; upper right; Fisher’s exact test; two-tailed *P* = 0.061).

## DISCUSSION

In this study, we assessed the inhibitory effects on virus replication, both *in vitro* and in the Syrian hamster model, of an array of drugs that are approved for other diseases. We evaluated only one dose and route for each drug, which we selected based on previous studies using rodent models to examine treatments for other pathogens or diseases (17, 32–41), or by using a dose conversion calculation from human to animal dose based on body weight; accordingly, this is a limitation of this study, because other dosages may result in different efficacy. Although some of the drugs did not show an effect *in vitro* and/or in the hamster model, the information obtained here may still be important for future drug candidate analyses for pandemic viruses.

Here, we showed that administration of favipiravir, either prophylactically or therapeutically, inhibits SARS-CoV-2 replication in the hamster infection model. Favipiravir is a prodrug of a nucleotide analog which interferes with the RNA-dependent RNA polymerase, acting as a pseudo purine (42, 43). Favipiravir was first approved as an anti-influenza virus drug in Japan, and since then has been shown to be effective against many other RNA viruses, including Ebola virus, Lassa virus, and Sudan virus in animal models (44–47); this suggests its broad-spectrum and more general utility against RNA viruses, including novel viruses that may emerge in the future.

A recent pharmacokinetic study showed that favipiravir at a single dose of 25 mg/animal, which is comparable to the dosing used in our study (24 to 28 mg/animal/administration based on body weight), gave concentrations of 135 μg/mL and 81.3 μg/g in the plasma and lungs, respectively, at 5 h post-administration, although the concentration in plasma exponentially decreased to 1.43 μg/mL at 12 h post-administration (13). Based on the EC_50_ values in our study (130 μM and 320 μM in VeroE6/TMPRSS2 cells and Calu-3 cells, respectively; corresponding to 20.4 μg/mL and 50.3 μg/mL, respectively) and the EC_50_ value of 61.88 μM (corresponding to 9.7 μg/mL) with VeroE6 cells from a previous study (21), the *in vivo* effectiveness in hamsters and that of *in vitro* assays seems to be consistent. Notably, in clinical trials, favipiravir administration to humans (1,800 mg twice/day on day 1 followed by 800 mg twice/day for 13 days) resulted in mean daily trough levels of >20 μg/mL without causing remarkable adverse effects (48). Pharmacokinetics in humans differ from those in hamsters, and favipiravir levels in human respiratory organs remain to be examined further; however, these results suggest that favipiravir treatment could be a safe and feasible option for COVID-19 therapy.

Remdesivir is also a broad-spectrum prodrug of a nucleotide analog. In terms of COVID-19 treatment, the WHO Solidarity trial found that remdesivir had little to no effect on mortality in hospitalized patients when administered late (49); however, several randomized clinical trials have suggested that remdesivir can shorten recovery time and/or improve prognosis (50). Also, notably, remdesivir treatment of COVID-19 patients with deficient humoral immunity reduced sub-genomic viral RNA levels in their respiratory tracts by several log-fold, thereby demonstrating its *in vivo* efficacy in humans (51). Although its antiviral effects on SARS-CoV-2 and Ebola virus have been shown in nonhuman primate models (52, 53), remdesivir did not significantly reduce SARS-CoV-2 replication in our hamster model; this may be partly explained by the previous finding that remdesivir is rapidly metabolized in rodents due to high serum esterase activity (54, 55). In contrast, Yuan et al. reported that intraperitoneal administration of remdesivir (15 mg/kg) in a therapeutic regimen reduced viral load in the lungs, underscoring the importance of testing drugs in several different animal models and using different administration routes (15). We did, however, find that GS-441524, a metabolite of remdesivir, effectively inhibited SARS-CoV-2 replication in hamsters. GS-441524 is currently available only as a research compound; however, its safety and effectiveness have been experimentally proven for treating feline infectious peritonitis caused by feline coronavirus infection (28, 29). In previous studies, GS-441524 was administered to cats via the intravenous and/or subcutaneous route (28, 29), and remdesivir was administered intravenously to nonhuman primates (52, 53). Therefore, in this study, the drugs were subcutaneously administered to hamsters. However, a recent pharmacokinetic study in mice demonstrated that oral or intravenous administration of GS-441524 led to comparable GS-441524 plasma levels and resulted in distribution of the drug in the lungs and liver (56). Of note, a phase I clinical trial of orally administered GS-441524 for COVID-19 therapy has been reported in July 2021, although the data are preliminary and the number of participants in the study was limited (https://clinicaltrials.gov/ct2/show/study/NCT04859244; https://osf.io/am5s8/ as a preprint manuscript). These findings support the feasibility of GS-441524 administration to humans. Both remdesivir and GS-441524 are detectable in plasma after administration and are cell-membrane permeable, whereas the active form that they are converted to (i.e., the triphosphate analog GS-443902) exists only within cells (57). Recent studies have shown that GS-441524 has inhibitory potency comparable to that of remdesivir on SARS-CoV-2 replication in several cell lines, including VeroE6 cells and human primary cells (23, 58), and that its antiviral activity in those cells correlates with the intracellular concentration of GS-443902 converted from either remdesivir or GS-441524 (23). Moreover, recent pharmacokinetic and pharmacodynamic studies of remdesivir in humans showed that the concentration of intravenously injected remdesivir declines rapidly, with a half-life of ∼1 h in plasma, followed by an increase in GS-441524 concentration that persists for much longer, with a half-life range of 13 to 31 h (57, 59, 60). These findings suggest that GS-441524 shows promise as a therapeutic option to treat SARS-CoV-2 infection.

In this study, we found that co-administration of favipiravir and GS-441524 effectively suppressed SARS-CoV-2 replication in hamsters, reducing the viral load in the lungs by 2 log-fold with low (10 PFU) or high (1,000 PFU) titer virus challenge, although the titers in the nasal turbinate were less affected. Under some experimental conditions, the inhibitory effects of dosage on virus replication were more prominent with the high-titer virus challenge (i.e., virus loads in the lungs with favipiravir therapeutic administration [Fig. 1B]; in the nasal turbinates with favipiravir/GS-441524 therapeutic administration [Fig. 3B]). We previously observed that a SARS-CoV-2 inoculation of 10 PFU/animal was sufficient for the virus to replicate efficiently to reach titers similar to those seen in animals infected with 1,000 PFU, and that the growth kinetics were different depending on the challenge titers (Fig. S6), which may partly explain the apparent contradiction. The virus load reductions in the lungs and nasal turbinates with favipiravir administration (i.e., favipiravir therapeutic administration and 1,000 PFU of challenge virus [Fig. 1B and C; Fig. 3C and D]) varied to some extent; this variation might be due to the small sample size, however, the mean fold reduction was 1.09 log-fold and 1.32 log-fold in the lungs (Fig. 1B and Fig. 3C) and 1.06 log-fold and 1.47 log-fold in the nasal turbinates (Fig. 1C and Fig. 3D). Therefore, the difference between the experiments was not substantial.

10.1128/mBio.03044-21.9 FIG S6 SARS-CoV-2 replication in the lungs and nasal turbinate of hamsters inoculated with different infectious doses. Each group of hamsters was intranasally inoculated with 1, 10, 100, 1,000, or 100,000 PFU of SARS-CoV-2 (day 0). Infectious virus titers in the lungs and nasal turbinate (PFU/g) on days 3 and 6 post-inoculation were examined by performing plaque assays (*n* = 4/group on each day). Dashed lines show the detection limits in each assay (10 PFU/g). Download FIG S6, TIF file, 0.7 MB. Copyright © 2022 Chiba et al. 2022 Chiba et al. https://creativecommons.org/licenses/by/4.0/ This content is distributed under the terms of the Creative Commons Attribution 4.0 International license.

In this study, body weight change of infected animals was monitored as a clinical marker. However, some caution is required when interpreting these results because, in drug administration models, body weight is affected both by the disease severity caused by the virus infection and by the toxicity of the drug(s); in fact, a previous study has shown that high-dose favipiravir administration causes weight loss in uninfected hamsters (13). As a result, body weight loss is not necessarily correlated with virus titers in organs (Fig. 3C–E; Fig. S2–4). Also, body weight loss may not be obvious with lower challenge doses in the hamster SARS-CoV-2 infection model, and the extent of the weight loss may differ between experiments. Administration of the vehicle control to infected animals also may cause variations in body weight changes, depending on the route and amount administered. Pathological analyses in organs would be preferable to evaluate the severity of the disease more precisely.

Immunohistochemistry analyses revealed no difference between the numbers of nucleocapsid protein-positive bronchial and alveolar epithelial cells in the vehicle dosage group and those in the the favipiravir/GS-441524 prophylactic/therapeutic co-administration groups (Fig. 4I–K), whereas the infectious virus load in the lungs was reduced by 2 log-fold with either the prophylactic or therapeutic regimen compared to that of the vehicle dosage group (Fig. 3A). In contrast, the amount of viral RNA detected by *in situ* hybridization in the lungs was reduced with co-administration treatment for either regimen (Fig. 4E–G), demonstrating a better correlation with virus loads in this organ (Fig. 3A). Intriguingly, prophylactic co-administration more effectively reduced inflammatory cell infiltration into the lungs than did the therapeutic regimen (Fig. 4A and B), even though the infectious virus loads in the lungs were similar (Fig. 3A). These results suggest that pretreatment with these drugs may reduce inflammatory responses in the lungs; further studies would help elucidate the underlying mechanism.

Finally, we showed that in the hamster transmission model, co-administration of GS-441524 and favipiravir to animals prior to exposing them to infected animals provided partial protection from virus airborne transmission (4 of 6 pairs were protected, compared with none of the 6 pairs for the vehicle control group), suggesting the potential of co-administration as a preventive measure. The virus titers in the upper respiratory tract (nasal turbinate) in unprotected animals were not as reduced as those in the lungs (Fig. 5C), suggesting that those animals may emit viruses for further transmission. Nevertheless, based on our pathological analyses (Fig. 4), prophylaxis with these drugs should be highly effective at reducing disease severity in unprotected animals. Currently, GS-441524 is only approved for research use; however, as mentioned previously, a phase I clinical trial of GS-441524 as an oral therapy has been recently reported (https://clinicaltrials.gov/ct2/show/study/NCT04859244) which demonstrated that oral administration of GS-441524 to humans achieved better persistence of the drug concentration in plasma compared to IV administration of remdesivir; this suggests that oral administration of GS-441524 could be an option for SARS-CoV-2 treatment in the future. As for parenteral administration of the drug to naive contacts, some situations may be anticipated such as pretreatment of uninfected individuals with cluster infections in nursing homes or hospitalized individuals at high risk of infection due to immunodeficiency.

In conclusion, our findings with the hamster model in this study suggest that the repurposing and co-administration of approved drugs may be an attractive approach to combat COVID-19.

## MATERIALS AND METHODS

### Cells.

VeroE6/TMPRSS2 (JCRB 1819) cells were propagated in the presence of 1 mg/mL Geneticin (G418; InvivoGen) and 5 μg/mL plasmocin prophylactic (InvivoGen) in Dulbecco modified Eagle’s medium containing 10% fetal calf serum (FCS) and antibiotics. Calu-3 (ATCC HTB-55) cells were maintained in Eagle’s minimal essential media (MEM) containing 10% fetal calf serum (FCS) and antibiotics. Cells were incubated at 37°C with 5% CO_2_ and regularly tested for mycoplasma contamination using PCR, and were confirmed to be mycoplasma-free.

### Virus.

SARS-CoV-2 (SARS-CoV-2/UT-NCGM02/Human/2020/Tokyo) was propagated in VeroE6 cells in Opti-MEM I (Invitrogen) containing 0.3% bovine serum albumin and 1 μg of L-1-Tosylamide-2-phenylethyl chloromethyl ketone (TPCK)-trypsin/mL in MEM supplemented with 2% FCS at 37°C. The furin cleavage site sequence was confirmed to be intact by deep sequencing of the virus stock (data not shown). SARS-CoV-2 Spike-D614G variant was generated using reverse genetics based on the SARS-CoV-2 WA1 strain at Baric’s lab (31), and was propagated as described above.

### Compounds.

Remdesivir (GS-5734; cat no. 329511), nafamostat (cat. no. 329453), and GS-441524 (cat. no. 555299) were purchased from MedKoo Biosciences. Nelfinavir mesylate (cat. no. S4282) and Ciclesonide (cat. no. S4646) were purchased from Selleckchem. Lopinavir/ritonavir (80 mg:20 mg/mL; cat. no. 6250101S1035) was purchased from Abbvie. Hydroxychloroquine sulfate (cat. no. 1327000) was purchased from United States Pharmacopeia. Ivermectin (cat. no. PHR1380) came from Sigma. Mefloquine was purchased from Hisamitsu Pharmaceutical. Umifenovir hydrochloride (cat. no. HY-14904A) came from MedChemExpress. Cepharanthine came from Kaken Pharmaceutical. Favipiravir was a generous gift from FUJIFILM Toyama Chemical.

### Animal experiments.

All experiments with hamsters were performed in accordance with the Science Council of Japan’s Guidelines for Proper Conduct of Animal Experiments and the guidelines set by the Institutional Animal Care and Use Committee at the University of Wisconsin-Madison. The protocol was approved by the Animal Experiment Committee of the Institute of Medical Science, University of Tokyo (approval no. PA19-75) and the Animal Care and Use Committee of the University of Wisconsin-Madison (protocol no. V00806).

### Dosage and virus infection of Syrian hamsters.

Four-to-six-week-old Syrian hamsters (female or male) were purchased from Envigo (Indianapolis, IN, USA) or Japan SLC, Inc. (Shizuoka, Japan). For oral administration, compounds were suspended in 0.5% methylcellulose in water and were administered via plastic feeding tubes (18G, 50 mm) with an administration volume of 10 mL/kg. For subcutaneous inoculation, remdesivir and GS-441524 were suspended in 12% SBE-β-CD in water. For intranasal administration, ciclesonide (100 mg/mL) in 100% ethanol was diluted in saline at 1:100 and administered at a volume of 1 mL/kg. For intraperitoneal administration, nafamostat was suspended at 3 mg/mL in saline with an administration volume of 10 mL/kg. Under anesthesia by ketamine-xylazine or isoflurane inhalation, four hamsters per group were intranasally inoculated with 10 or 1,000 PFU/animal of SARS-CoV-2/UT-NCGM02/Human/2020/Tokyo. Body weights were monitored daily after infection. On day 4 postinfection, animals were euthanized and their lung lobes and nasal turbinates were pooled to determine virus titers by use of plaque assays in VeroE6/TMPRSS2 cells.

### Determination of half-maximal effective concentration values.

VeroE6/TMPRSS2 cells or Calu-3 cells were seeded in 96-well plates 1 day prior to infection, and were incubated at a multiplicity of infection (MOI) of 0.01 or 0.1, respectively, with SARS-CoV-2 at 37°C for 1 h. The inocula were then replaced with MEM containing 5% FCS and serially diluted drugs, and the cells were incubated at 37°C with 5% CO_2_ for 2 days to observe cytopathic effects (CPE).

### Determination of half-maximal inhibitory concentration values.

VeroE6/TMPRSS2 cells were seeded in 6-well plates 1 day prior to infection, and were incubated with 50 PFU/well of SARS-CoV-2 at 37°C for 1 h. After the inocula were aspirated, 2 mL/well of MEM containing 5% FCS and 1% agarose was layered onto the cells. After incubation at 37°C with 5% CO_2_ for 2 days, the cells were fixed with 10% formalin-PBS (phosphate-buffered saline) and the plaques were counted.

### Drug combination study.

VeroE6/TMPRSS2 cells were seeded in 6-well plates 1 day prior to infection. The cells were infected with virus diluent that resulted in 50 to 100 virus plaques per well. After incubation at 37°C for 1 h, the viral inoculum was removed and the cells were overlaid with MEM containing 1% of agarose and each drug-concentration combination. Relative inhibition of plaque formation (%) was calculated based on plaque number reduction compared that of the well without drugs. A ZIP synergy score was calculated and mapped using the SynergyFinder (v.2.0) application (30).

### Pathological examination.

Excised lung tissues were fixed in 10% formalin in PBS and processed for paraffin embedding. The paraffin blocks were cut into 3-μm-thick sections and then mounted on silane-coated glass slides. One section from each tissue sample was stained using a standard hematoxylin and eosin procedure; other sections were processed either for *in situ* hybridization using an RNA scope 2.5 HD Red Detection kit (Advanced Cell Diagnostics, Newark, California) with an antisense probe targeting the nucleocapsid gene of SARS-CoV-2 (Advanced Cell Diagnostics) or for immunohistochemical staining with a rabbit polyclonal antibody for SARS-CoV virus nucleocapsid protein (Prospec; ANT-180). Specific antigen-antibody reactions were visualized by means of 3,3′-diaminobenzidine tetrahydrochloride staining using the Dako Envision system (Dako Cytomation). To evaluate comprehensive histopathological changes, lung tissue sections were scored based on pathological changes. Scores were determined based on the percentage of inflammation area for each section of the five lobes collected from each animal in each group by using the following scoring system: 0, no pathological change; 1, affected area (≤10%); 2, affected area (<50%, >10%); 3, affected area (≥50%). An additional point was added when pulmonary edema and/or alveolar hemorrhage was observed. The total score for the five lobes is shown for each animal in Table S3.

### Virus transmission study in Syrian hamsters.

Naive hamsters (exposed animals) were administered favipiravir (300 mg/kg) and/or GS-441524 (25 mg/kg) or vehicle control twice/day (days –1 to 3 post-exposure; *n* = 6/group); another group of animals (infected animals) was inoculated with 1,000 PFU of SARS-CoV-2 Spike-D614G virus. One day after initiating drug treatment of the exposed animals and virus inoculation of the infected group, each exposed animal was paired with one of the infected animals with a 5-cm gap between their cages in an isolator unit (Showa Science). Air supply and exhaust for each isolator unit was filtered by high efficiency particulate air (HEPA) filters to prevent cross-contamination, resulting in unidirectional airflow directed from the ‘infected animal’ to the ‘exposed animal’ in each isolator unit. The infected animals and the exposed animals were euthanized to titrate the virus loads in the nasal turbinate and lungs on day 4 postinfection and day 4 post-exposure, respectively. For virus titration, whole lobes of the lungs were harvested, pooled, weighed, and homogenized. For each lung or nasal turbinate sample, 1 mL of medium was added and, after homogenization in the media, 100 μL of the homogenate was subjected to titration in plaque assays. The detected plaque numbers were then divided by the weight of the organ. The detection limit of the virus in this study was 10 PFU/organ.

### Statistics.

Statistical analysis was performed in GraphPad Prism 8 using a one-way ANOVA corrected by using Dunnett’s test for multigroup comparison or an unpaired *t* test for two groups.

### Data availability.

All study data are included in the article.
