# Supplementary material for: Co-administration of Favipiravir and the Remdesivir Metabolite GS-441524 Effectively Reduces SARS-CoV-2 Replication in the Lungs of the Syrian Hamster Model
Source: mBio. 2022 Feb 1;13(1):e03044-21. doi: 10.1128/mbio.03044-21 (PMC8805032; doi:10.1128/mbio.03044-21)
Supplement: TABLE S1 [file mbio.03044-21-st001.docx]

**Table S1. Dosage regimens in Syrian hamster models**

|  | Route* | Dose | Regimen** |
| --- | --- | --- | --- |
| Favipiravir (prophylactic) | p.o. | 300 mg/kg | Twice/day (Day –1 to 3) |
| Favipiravir (therapeutic) | p.o. | 300 mg/kg | Twice/day (Day 1 to 3) |
| Lopinavir/Ritonavir | p.o | 80 mg:20 mg/kg | Twice/day (Day –1 to 3) |
| Nelfinavir | p.o | 125 mg/kg | Twice/day (Day –1 to 3) |
| Hydroxychloroquine sulfate | p.o | 20 mg/kg | Twice/day (Day –1 to 3) |
| Remdesivir (GS-5734) | s.c. | 25 mg/kg | Twice/day (Day –1 to 3) |
| Ciclesonide | i.n. | 1 mg/kg | Once/day (Day –1 to 3) |
| Nafamostat mesylate | i.p. | 30 mg/kg | Once/day (Day –1 to 3) |
| Ivermectin | p.o. | 2 mg/kg | Single shot (Day –1) |
| Mefloquine | p.o. | 20 mg/kg | Single shot (Day –1) |
| Umifenovir | p.o. | 25 mg/kg | Twice/day (Day -1 to 3) |
| Cepharanthine | p.o. | 0.05 mg/kg | Twice/day (Day –1 to 3) |
| GS-441524 (prophylactic) | s.c. | 25 mg/kg | Twice/day (Day –1 to 3) |
| GS-441524 (therapeutic) | s.c. | 25 mg/kg | Twice/day (Day 1 to 3) |

* p.o.: oral gavage; s.c.: subcutaneous inoculation; i.n.: intranasal inoculation; i.p.: intraperitoneal inoculation

** The animals were infected with virus on Day 0
