# Supplementary figures and images for: Co-administration of Favipiravir and the Remdesivir Metabolite GS-441524 Effectively Reduces SARS-CoV-2 Replication in the Lungs of the Syrian Hamster Model
Source: mBio. 2022 Feb 1;13(1):e03044-21. doi: 10.1128/mbio.03044-21 (PMC8805032; doi:10.1128/mbio.03044-21)

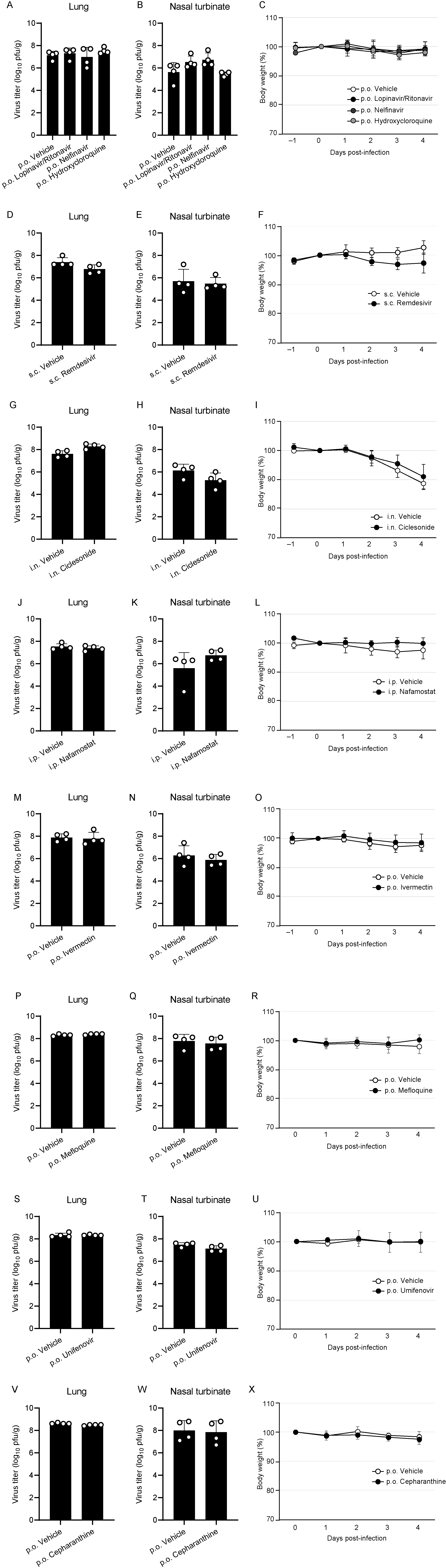

Supplement: FIG S1 [file mbio.03044-21-sf001.tif]

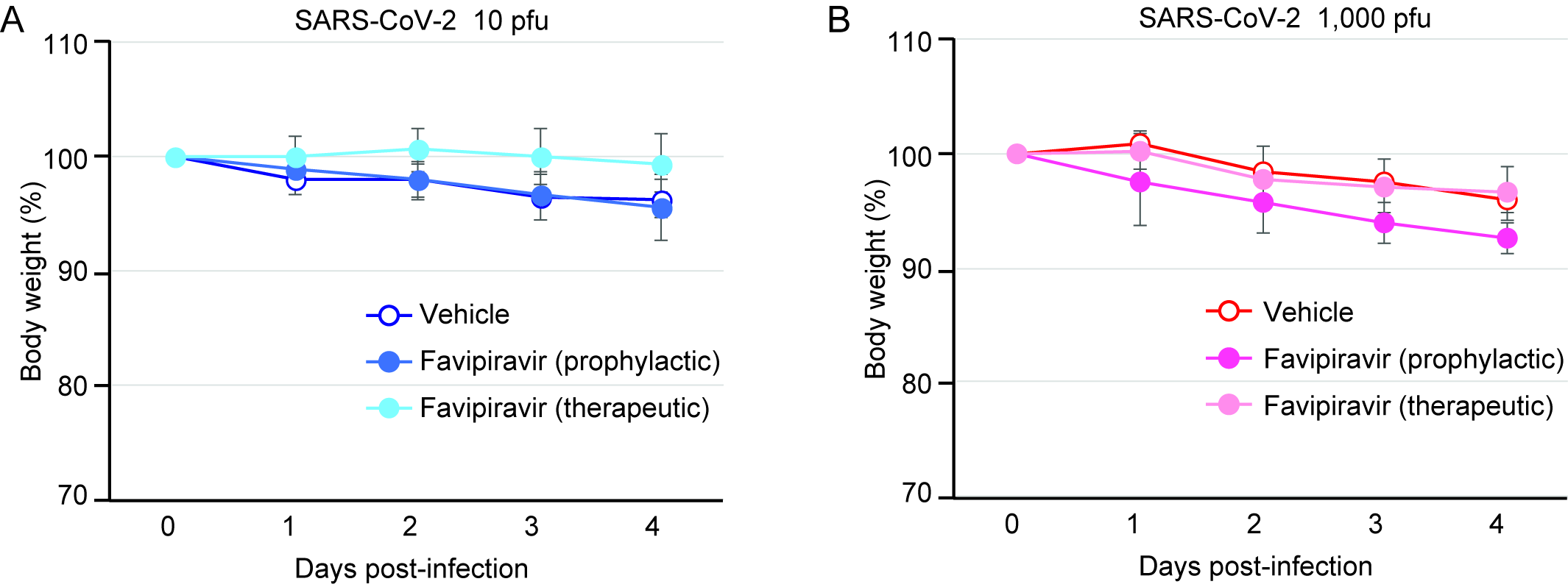

Supplement: FIG S2 [file mbio.03044-21-sf002.tif]

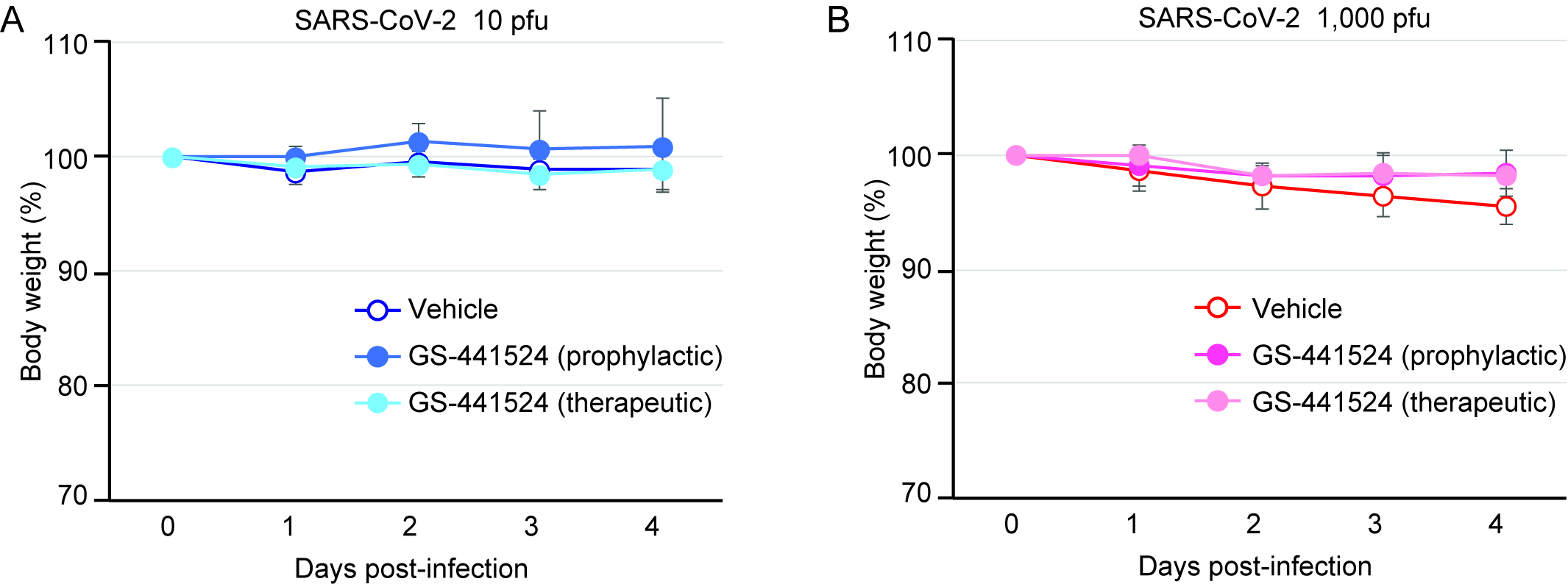

Supplement: FIG S3 [file mbio.03044-21-sf003.tif]

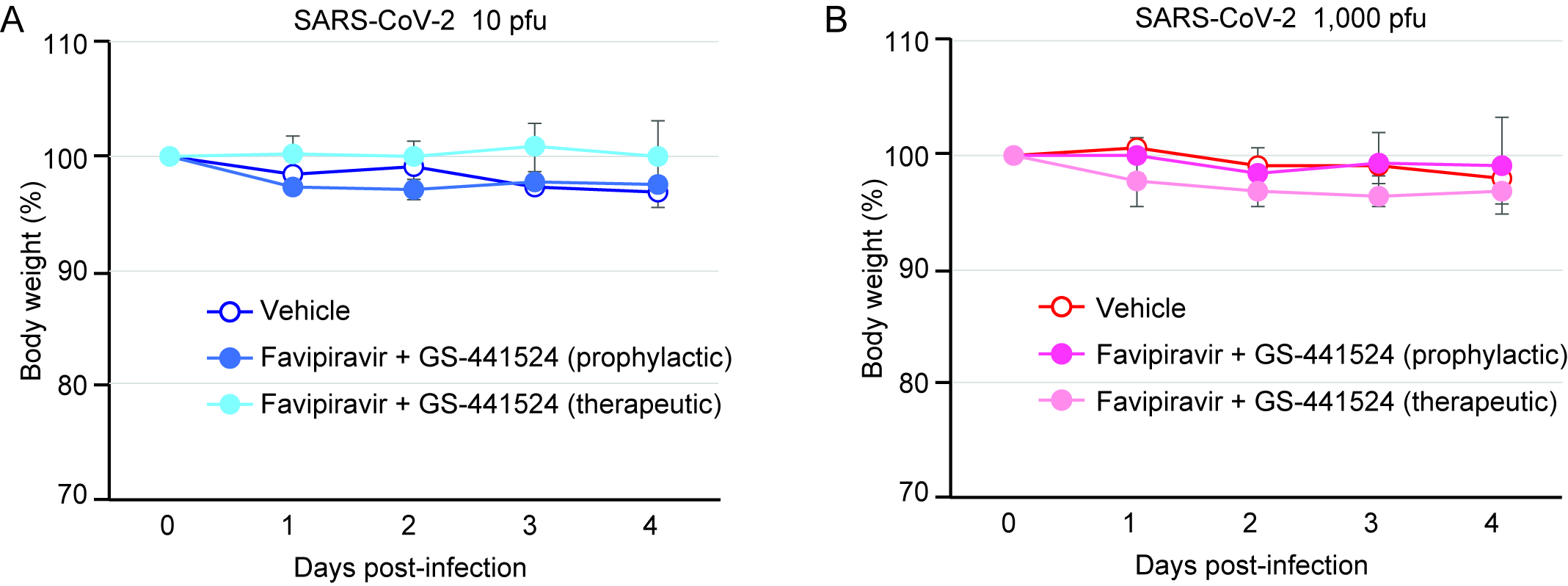

Supplement: FIG S4 [file mbio.03044-21-sf004.tif]

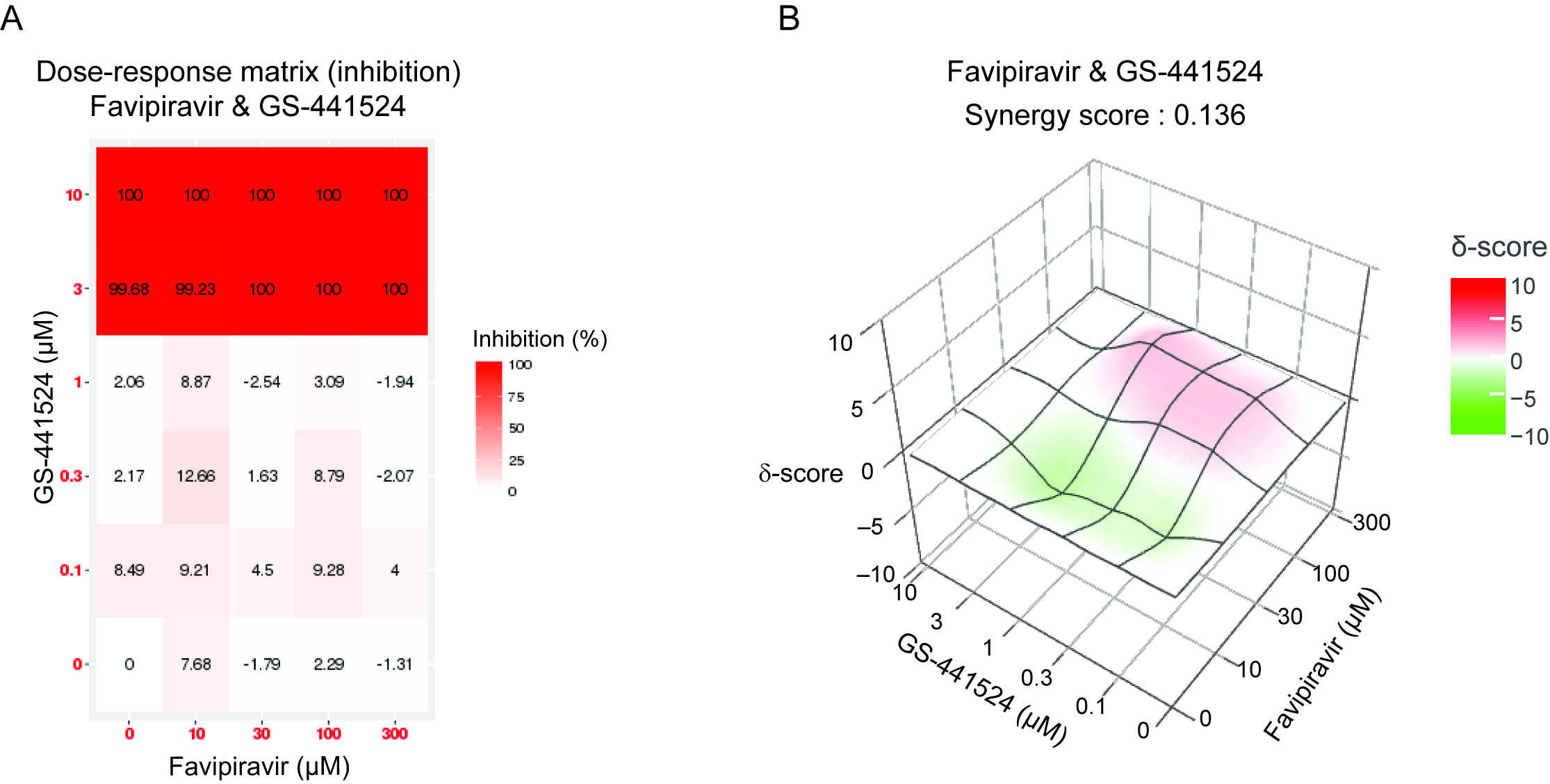

Supplement: FIG S5 [file mbio.03044-21-sf005.tif]

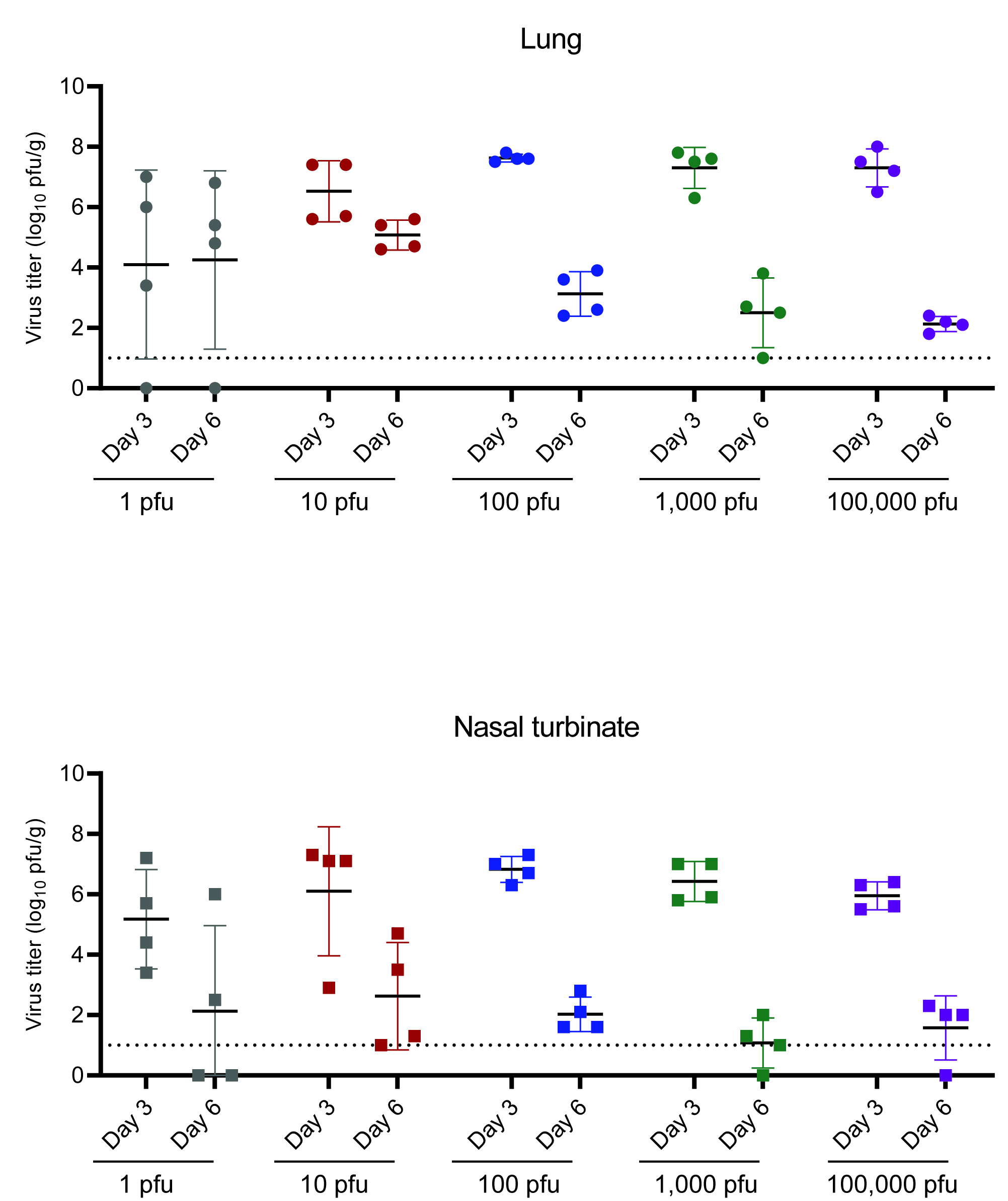

Supplement: FIG S6 [file mbio.03044-21-sf006.tif]
