# Supplementary material for: Co-administration of Favipiravir and the Remdesivir Metabolite GS-441524 Effectively Reduces SARS-CoV-2 Replication in the Lungs of the Syrian Hamster Model
Source: mBio. 2022 Feb 1;13(1):e03044-21. doi: 10.1128/mbio.03044-21 (PMC8805032; doi:10.1128/mbio.03044-21)
Supplement: TABLE S2 [file mbio.03044-21-st002.docx]

**Table S2. Inhibitory effects of drugs on SARS-CoV-2 replication *in vitro***

|  | EC_50_ (μM) ** | | | IC_50_ (μM) ** |
| --- | --- | --- | --- | --- |
|  | | **VeroE6/TMPRSS2** | **Calu-3** | **VeroE6/TMPRSS2** |
| Favipiravir | 130 | | 320 | >100* |
| Lopinavir | 5.5 | | 3.2 | 12.2 |
| Ritonavir | >100* | | >100* | >100* |
| Nelfinavir | 5.5 | | 0.3 | 5.4 |
| Hydroxychloroquine sulfate | 54.8 | | 32 | 40 |
| Remdesivir (GS-5734) | 1.7 | | 0.3 | 9.7 |
| Ciclesonide | 17.3 | | >10* | 13.9 |
| Nafamostat mesylate | >100* | | 3 | >100* |
| Ivermectin | >30* | | 32 | >30* |
| Mefloquine | >100* | | >100* | >30* |
| Umifenovir | >100* | | >100* | >100* |
| Cepharanthine | >100* | | >100* | >100* |

*Concentration could not be determined at higher concentrations because of cytotoxicity

** EC_50_ : Cytopathic effect (CPE) inhibition assay; IC_50_ : plaque reduction assay
