# Supplementary material for: Co-administration of Favipiravir and the Remdesivir Metabolite GS-441524 Effectively Reduces SARS-CoV-2 Replication in the Lungs of the Syrian Hamster Model
Source: mBio. 2022 Feb 1;13(1):e03044-21. doi: 10.1128/mbio.03044-21 (PMC8805032; doi:10.1128/mbio.03044-21)
Supplement: TABLE S3 [file mbio.03044-21-st003.docx]

**Table S3. Pathological severity scores of the lung tissue sections**

| Animal ID# | Virus infection | Drug administration | Lobe 1 | Lobe 2 | Lobe 3 | Lobe 4 | Lobe 5 | Total score |
| --- | --- | --- | --- | --- | --- | --- | --- | --- |
| #13 | + | Prophylactic | 0 | 0 | 1 | 1 | 0 | 2 |
| #14 | + |  | 1 | 1 | 2 | 2 | 2 | 8 |
| #15 | + |  | 1 | 1 | 1 | 1 | 1 | 5 |
| #16 | + | Therapeutic | 4 | 2 | 4 | 2 | 4 | 16 |
| #17 | + |  | 2 | 2 | 3 | 4 | 3 | 14 |
| #18 | + |  | 4 | 2 | 3 | 2 | 3 | 14 |
| #19 | + | Vehicle | 4 | 1 | 3 | 3 | 3 | 14 |
| #20 | + |  | 4 | 3 | 3 | 3 | 3 | 16 |
| #21 | + |  | 4 | 2 | 4 | 2 | 4 | 16 |
| #22 | – | Vehicle | 0 | 0 | 0 | 0 | 1 | 1 |

Scoring system: 0, no pathological change; 1, affected area (≤10%); 2, affected area (<50%, >10%); 3, affected area (≥50%); an additional point was added when pulmonary edema and/or alveolar hemorrhage was observed.
